# Percutaneous Approach in Endovascular Aortic Procedures Using a Suture-Mediated Closure Device

**DOI:** 10.3390/jcm11226660

**Published:** 2022-11-10

**Authors:** Kyriakos Oikonomou, Akaki Kvataia, Karin Pfister, Evgenia Zygouridou, Thomas Betz, Wilma Schierling, Georgios Sachsamanis

**Affiliations:** 1 Department of Vascular and Endovascular Surgery, University Medical Center Regensburg, Franz-Josef-Strauss-Allee 11, 93053 Regensburg, Germany; 2 Department of Vascular and Endovascular Surgery, Cardiovascular Surgery Clinic, University Hospital Frankfurt, Johann Wolfgang Goethe University Frankfurt, 60596 Frankfurt, Germany

**Keywords:** ProGlide, suture-mediated closure device, endovascular, aortic repair, percutaneous

## Abstract

Background: The purpose of this study is to assess the efficacy of a suture-mediated closure device during percutaneous endovascular aortic repair. Methods: A single-center, retrospective analysis of patients undergoing endovascular repair for infrarenal, thoracic and thoracoabdominal aortic aneurysms and aortic dissections via percutaneous femoral access between April 2017 and June 2021 was performed. The primary endpoint of the study was the efficacy and technical success of the Perclose ProGlide closure device during percutaneous endovascular procedures. The secondary endpoints were intraoperative and postoperative inguinal and vascular complications during and after device use. Results: A total of 376 punctures were performed in 263 patients with the deployment of the ProGlide vascular closure system. Twenty-two cases involved percutaneous re-puncture as part of a staged procedure. The primary and secondary technical success rates were 93.1% (350/376) and 94.7% (356/376), respectively. In 20 patients (5.3%), intraoperative femoral exposure due to complications was required. Postoperative complications occurred in 13 cases (3.5%), 2 of which required surgical reintervention. There was no statistical significance between the type of endovascular procedure and primary technical success (*p* = 0.56). The introduction of larger-diameter sheaths was not associated with increased intraoperative and postoperative complication rates (*p* = 0.75 and *p* = 0.78, respectively). Percutaneous re-puncture of the vascular access site did not result in a lower overall technical success rate (20/22, 90.9% primary technical success rate, *p* = 0.67; 21/22, 95.5% secondary technical success rate, *p* = 0.86) or an increased number of perioperative complications (1/22, 4.5% intraoperative complications, *p* = 0.86; 2/22, 9.1% postoperative complications, *p* = 0.13). Conclusion: The application of the ProGlide closure system is a safe and efficient method to achieve hemostasis during percutaneous endovascular aortic repair. Complex aortic pathologies, which often require a staged approach with re-puncture, can also be successfully treated with this closure system.

## 1. Introduction

Surgical cut-down (SCD) femoral access has been widely used during endovascular aortic repair in the last 20 years [1]. It provides direct visualization of the vessel, allowing direct vascular access and confirming hemostasis on the table at the end of the procedure. It is, however, associated with a number of access-site complications, such as infection, the formation of lymphoceles and seromas [2]. A report from Vierhout et al. demonstrated a 9.5% complication rate for the open-access group (13/137 patients) [3]. In another recent report from Rebelo et al., surgical cut-down access during EVAR was associated with a 9% complication rate (20/215 patients) [4].

A number of arteriotomy closure devices (ACD) are commercially available, allowing complete percutaneous vascular access and bleeding control after the transfemoral introduction of large-diameter sheaths. Their application reduces operation time and length of stay and minimizes the risk of postoperative complications [3,5]. In addition, recent studies suggest that percutaneous access during endovascular aortic repair has lower costs in comparison to femoral exposure [6]. Although most data are in favor of ACD, there are a number of recent reports demonstrating average results and questioning the superiority of closure devices over SCD femoral access [7,8].

In this context, the aim of this study is to present our experience with percutaneous access during endovascular aortic procedures using the Perclose ProGlide^TM^ suture-mediated closure device (SMCD) from Abbott.

## 2. Methods and Materials

We conducted a single-center, retrospective analysis of patients who underwent endovascular repair for infrarenal, thoracic and thoracoabdominal aortic aneurysms as well as aortic dissections between April 2017 and June 2021. During this period, no patients planned for endovascular aortic repair were primarily excluded from percutaneous femoral access. 

Hemostasis at the end of the procedure was achieved using a ‘preclosure’ technique with the Perclose ProGlide system (Abbott Vascular, Abbott Park, IL, USA). Ultrasound-guided puncture was implemented to achieve vessel access under the supervision of experienced operators. In cases of extensive femoral calcification, the distal external iliac artery was punctured to achieve access. In cases where catheter insertion of up to 8 French was planned, one ProGlide system was applied in order to achieve hemostasis at the end of the procedure. In cases of larger-diameter sheaths, two closure systems were used at an angle of 60°. Additional closure systems were used for device deployment failures, or at the end of the procedure in cases of suture deployment failure or persistent bleeding. Vessel patency and the inguinal region were controlled at the end of the procedure and during the 1st postoperative day using duplex ultrasound. The ethics committee of our institution has approved this study (registration number 12-121-4-101).

### 2.1. Closure Device

The Perclose ProGlide closure device is a suture-mediated closure device designed to deliver a single, non-masking monofilament polypropylene suture in order to achieve hemostasis following arterial puncture during diagnostic or interventional catheterization procedures. It tracks over a standard 0.035″ or smaller guidewire, featuring a valve to achieve hemostasis during suture placement. A knot pusher allows for the positioning of the tied suture to the top of the arteriotomy at the end of the procedure. One to three closure systems in angles from 30 to 60 degrees can be deployed during vessel access in order to achieve hemostasis after the introduction of large-diameter sheaths of up to 21 French. It has no restrictions on re-access.

### 2.2. Endpoints

The primary endpoint of the study was the efficacy and technical success of the Perclose ProGlide closure device during percutaneous endovascular procedures. Primary technical success was defined as hemostasis after suture placement without an additional non-invasive or open surgical method during the completion of the procedure. Secondary technical success was defined as bleeding control after the placement of additional closure devices at the end of the procedure. The secondary endpoints were intraoperative and postoperative inguinal and vascular complications during and after device use, such as reoperations due to system failure, inguinal and retroperitoneal hematomas, pseudoaneurysms, distal embolization and vessel stenosis, occlusion or dissection. Additionally, all major and minor events during the postoperative period were collected and analyzed. Major events were defined as complications that led to reinterventions, while minor events were treated conservatively.

### 2.3. Statistical Analysis

The analysis of collected data was performed using SPSS for Windows (Version 27; SPSS INC, Chicago, IL, USA), with variables being presented as mean ± standard deviation (SD) in the case of a normal distribution and as median plus range when the data had a skewed distribution. Groups were compared with Chi-square, Fisher’s exact and Mann–Whitney U tests according to the type of data. Statistically analyzed data were considered significant when *p* < 0.05. 

## 3. Results

A total of 263 patients (200 males, 63 females, mean age 67.5 ± 10.4 years) underwent endovascular aortic procedures through percutaneous transfemoral access during the study period. Patient demographics and vessel characteristics are summarized in Table 1. A total of 136 patients underwent endovascular aortic aneurysm repair (EVAR) for infrarenal aortic aneurysm, 31 patients underwent thoracic EVAR, 35 patients underwent endovascular repair of a thoracoabdominal aneurysm (TAAA), and 61 patients were treated endovascularly for aortic dissection. A total of 376 punctures with the deployment of the ProGlide vascular closure system were performed. Access via the right and left common femoral arteries (CFAs) was obtained in 226 and 150 cases, respectively. A total of 113 patients had bilateral vascular access. None of the patients had a highly branched deep femoral artery. Vessel characteristics are presented in Table 2. In 22 cases, percutaneous re-puncture for endovascular access was obtained at sites where a ProGlide closure device had already been deployed. The interval between the first puncture and re-puncture was 105 ± 76 days. 

A total of 677 closure devices were used to achieve hemostasis at the end of the procedure: 414 ProGlide devices were used on the right CFA, and 263 were used on the left CFA. A total of 119 additional devices were used due to insufficient needle penetration through the vessel wall during deployment or suture break-up during vessel closure (68 (16.4%) and 51 (19.2%) on the right and left sides, respectively). The average sheath diameter was 15.15 ± 5.1 French (16.6 ± 4.9 and 12.8 ± 4.4 French on the right and left CFAs, respectively).

### 3.1. Technical Success

Primary technical success was 93.1% (350/376). In six cases, the deployment of an additional closure device was required at the end of the procedure to achieve adequate hemostasis, leading to a secondary technical success rate of 94.7% (356/376). Regarding the access site location, the primary and secondary technical success rates on the right CFA were 92.9% (210/226) and 95.1% (215/226), respectively, while on the left CFA, the primary and secondary technical success rates were 93.3% (140/150) and 94% (141/150), respectively. There was no statistical significance between peripheral artery disease (PAD), body mass index (BMI) and primary technical success (*p* = 0.78 and *p* = 0.26). Additionally, there was no statistical significance between PAD and perioperative complications (*p* = 0.75 and *p* = 1 for intraoperative and postoperative complications, respectively).

### 3.2. Intraoperative Complications

In 20 patients (5.3%), femoral exposure due to complications was required. In nine cases, persistent bleeding after the deployment of a third closure device at the end of the operation led to femoral artery exposure and open surgical reconstruction. In six cases, surgical cut-down access was needed after device failure during deployment. Two patients required open femoral reconstruction due to hemodynamically significant vessel stenosis after securing the sutures. Two other patients had a hemodynamically significant dissection of the CFA and the distal external iliac artery (EIA). In one case, the fixation of the closure device led to complete occlusion of the CFA, requiring open surgical exposure and arterial reconstruction with a biological patch. A summary of the intraoperative complications with regard to the access site location and the type of management is presented in Table 3. 

### 3.3. Postoperative Complications

Postoperative complications were seen in 13 cases (3.5%). Two patients had major complications requiring reintervention. One patient underwent surgical evacuation of a subcutaneous hematoma, while another patient underwent resection of a femoral pseudoaneurysm. Minor adverse events included a subcutaneous hematoma in six cases and the formation of a femoral pseudoaneurysm in two cases. Additionally, stenosis and dissection of the femoral artery and the formation of an arteriovenous fistula were observed in one patient each. None had a hemodynamically significant reduction in the extremity’s perfusion, and no reinterventions were required. A summary of the postoperative complications with regard to the access site location is presented in Table 4.

### 3.4. Endovascular Procedure and Sheath Diameter

**Figure 1:**
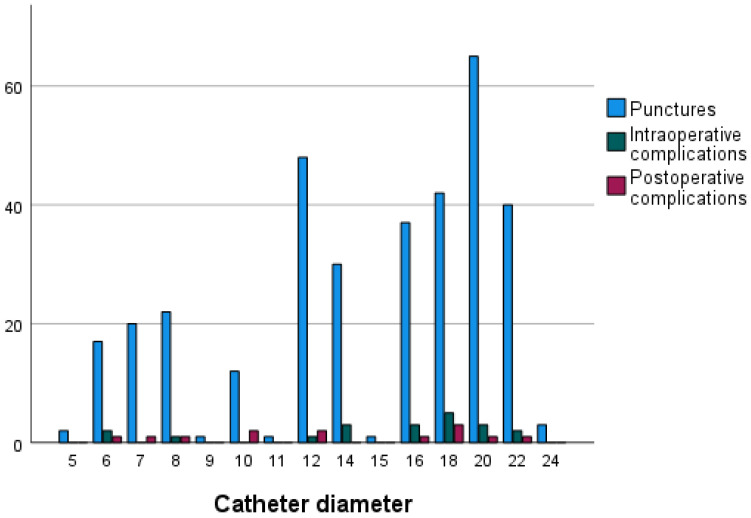
Number of intraoperative and postoperative complications with regard to introduced sheath diameter.

There was no statistical significance between the type of endovascular procedure and primary technical success (*p* = 0.56). The sheath diameter was not associated with technical failure (*p* = 0.69 for primary and *p* = 0.76 for secondary technical success). Further, the introduction of larger-diameter sheaths was not associated with increased intraoperative and postoperative complication rates (*p* = 0.75 and *p* = 0.78, respectively) (Figure 1).

### 3.5. Re-Puncture of Access Site

Due to the staged approach, a ProGlide SMD had already been used in 22 cases. No other closure device had been previously used. Percutaneous vascular access in these previously punctured femoral sites was not associated with a lower overall technical success rate (20/22, 90.9% primary technical success rate, *p* = 0.67; 21/22, 95.5% secondary technical success rate, *p* = 0.86). Furthermore, it was not associated with an increased number of perioperative complications (1/22, 4.5% intraoperative complications, *p* = 0.86; 2/22, 9.1% postoperative complications, *p* = 0.13). One patient exhibited persistent bleeding after suture placement, requiring the placement of a third closure device to achieve adequate hemostasis. In one case, there was a dissection of the CFA after securing the tied suture, leading to femoral exposure and arterial reconstruction. The postoperative complications in this subgroup included one local dissection and one pseudoaneurysm of the CFA, both of which were managed conservatively.

## 4. Discussion

There are a number of reports demonstrating conflicting results regarding the application of the ProGlide closure device to attain bleeding control after percutaneous endovascular aortic procedures. In one of the first multicenter randomized controlled trials (PEVAR), Nelson et al. compared the efficacy of two percutaneous closure devices (Prostar XL and ProGlide) and femoral exposure during EVAR. They achieved an 88% primary technical success in 50 patients using the ProGlide closure device, demonstrating significantly better results in comparison to femoral exposure (*p* = 0.004). The vascular complication rate was 8% [1].

In a large review by Karaolanis et al., technical success using the Prostar XL and ProGlide closure devices ranged between 63% and 100%, with only 2 articles reporting rates below 80% and 28 articles reporting technical success rates between 89% and 100%. The complication rates ranged between 0% and 25% [10].

In a recent single-center study, Mathisen et al. evaluated the efficacy of the ProGlide closure device for the closure of large-bore puncture holes after EVAR. They reported primary and secondary technical success rates of 68.1% and 96.9%, respectively. However, this was achieved with the additional application of the AngioSeal closure device. The authors recorded 31 complications in 837 groins (3.7%) [11]. They suggest that the low primary success rates may be a result of various physicians during the application of the device and the absence of ultrasound-guided puncture during the early stages of the study [11]. 

There were no primary exclusion criteria for percutaneous access in this patient cohort. The inclusion of patients with peripheral artery disease and previously punctured groins allows for a direct comparison between subgroups of patients who are usually considered unsuitable for a percutaneous approach. A study by Shen-Yen Lin et al. showed that anterior calcification of the common femoral artery (CFA) is a significant predictor of additional ProGlide deployment during EVAR [12]. Our study suggests that the presence of PAD does not seem to affect technical success.

Our study in a large series of patients demonstrates that the application of the ProGlide closure system after ultrasound-guided femoral puncture is a safe and effective method to achieve hemostasis during percutaneous endovascular aortic procedures. Primary (93.1%) and secondary (94.7%) technical success rates were high, and intraoperative (5.3%) and postoperative (3.5%) complication rates were low. In particular, avoiding the occurrence of lymphatic fistulas is a major advantage of percutaneous access. Before the introduction of percutaneous access, lymphatic fistulas occurred in approximately 12.5% of cases in our cohort (15/120 cases with open exposure of the femoral access site in the period 01/2015–03/2017). Patients undergoing complex endovascular repair for thoracoabdominal pathologies were also included in this series. The application of the ProGlide closure device also appears to be effective in the endovascular management of thoracoabdominal and thoracic aortic aneurysms, where the introduction and frequent exchange of large-diameter sheaths is required. In addition, the closure system can be effectively used in the endovascular repair of complex aortic pathologies requiring a staged endovascular approach and re-puncture of the vascular access site. Only 2 out of 22 sites with re-punctures had a primary technical failure with the closure device, and only 1 out of these 22 cases required surgical revision. 

The limitations of our study are its retrospective, nonrandomized and observational nature. Additionally, a cost–effect analysis was not performed. Prospective or multicenter randomized control trials are needed in order to better assess the efficacy of SMCDs compared with other closure techniques.

## 5. Conclusions

The use of the Perclose ProGlide^TM^ suture-mediated closure device is a safe and efficient method to achieve hemostasis during percutaneous endovascular aortic repair, even when large-diameter sheaths are introduced. Its deployment also appears to be safe during endovascular repair of complex aortic pathologies that require a staged endovascular approach and thus re-puncture of the femoral access site. In our experience, both ultrasound-guided puncture of the femoral vessels and intraoperative and postoperative monitoring of the access site are mandatory to minimize the complication rate and to allow immediate revision in the case of treatment failure or complications. 

## Figures and Tables

**Table 1:** Patient demographics. No. = number; y.o = years old; SD = standard deviation; BMI = Body Mass Index; ASA; American Society of Anesthesiology score; CAD = coronary artery disease; hypertension = blood pressure > 130/80 mmHg; COPD = chronic obstructive pulmonary disease: PAD = peripheral artery disease; diabetes = HbA1C > 7%; hypercholesterolemia = total cholesterol levels > 200 mg/dL; CKD = chronic kidney disease.

Variable	No. (%)
Male/female ratio	200 (76%)/63 (24%)
Mean patient age (y.o ± SD)	67.5 ± 10.4
BMI	27.4 ± 4.6
ASA Grading	
- ASA I	1 (0.4%)
- ASA II	34 (12.9%)
- ASA III	184 (70%)
- ASA IV	44 (16.7)
CAD	76 (28.9%)
Hypertension	237 (90.1%)
COPD	54 (20.5%)
PAD	40 (15.2%)
Diabetes	42 (16%)
Smoking	125 (47.5%
Hypercholesterolemia	190 (72.2%)
CKD	172 (65.4%)

**Table 2:** Vessel characteristics. CFA = common femoral artery; No. = number; SD = standard deviation; EIA = external iliac artery; DFA = deep femoral artery; SFA = superficial femoral artery; calcification levels according to Manunga et al. [9].

CFA Calcification	No. (%)
- None	264 (70.2%)
- <50% posterior	92 (24.4%)
- >50% posterior	9 (2.3%)
- <50% anterior	11 (2.9%)
- >50% anterior	0
Vessel diameter	mm ± SD
- EIA	93 ± 16
- CFA	92 ± 25
- DFA	71 ± 17
- SFA	74 ± 14

**Table 3:** Intraoperative complications requiring femoral exposure in relation to site access location and type of management. No. = number; CFA = common femoral artery.

Intraoperative Complications	No. (%)
Right CFA	11 (2.1%)
- Persistent bleeding	4 (0.8%)
- Deployment failure	4 (0.8%)
- Stenosis	1 (0.2%)
- Dissection	2 (0.4%)
- Occlusion	0 (0%)
Left CFA	9 (1.7%)
- Persistent bleeding	4 (0.8%)
- Deployment failure	3(0.6%)
- Stenosis	1 (0.2%)
- Dissection	0 (0%)
- Occlusion	1 (0.2%)
Type of management	
- Direct arterial reconstruction	16 (80%)
- Biological patch	3 (15%)
- Fascial closure	1 (5%)

**Table 4:** Postoperative complications in relation to site access location. No. = number; CFA = common femoral artery; AV = arteriovenous.

Postoperative Complications	No. (%)
Right CFA	7 (1.3%)
- Hematoma	3 (0.6%), 1 reintervention
- Pseudoaneurysm	2 (0.4%)
- Stenosis	1 (0.2%)
- AV fistula	1 (0.2%)
Left CFA	6 (1.1%)
- Hematoma	3 (0.6%)
- Pseudoaneurysm	1 (0.2%), 1 reintervention
- Stenosis	1 (0.2%)
- Dissection	1 (0.2%)

## Data Availability

The data presented in this study are available on request from the corresponding author. The data are not publicly available due to ethical and privacy compliance.
